# Minimum wiping pressure and number of wipes that can remove dirt during bed baths using disposable towels: a multi-study approach

**DOI:** 10.1186/s12912-022-01162-z

**Published:** 2023-01-16

**Authors:** Issei Konya, Kotone Nishiya, Inaho Shishido, Marie Hino, Kazuhiro Watanabe, Rika Yano

**Affiliations:** 1 Graduate School of Health Sciences, Hokkaido University, Sapporo, Japan; 2 Research Fellow of Japan Society for the Promotion of Science, Tokyo, Japan; 3 Faculty of Health Sciences, Hokkaido University, Sapporo, Japan

**Keywords:** Baths, Bed baths, Cross-sectional studies, Disposable wipes, Hygiene, Nursing care, Quasi-experimental studies, Skin care

## Abstract

**Background:**

Friction irritation by wiping increases the risk of skin problems. In bed baths with cotton towels, wiping three times with weak pressure (10–20 mmHg ≈ 1333–2666 Pa) can remove dirt while maintaining skin barrier function. However, few studies have examined the appropriate frictional irritation with disposable towels. This study aimed to analyse the wiping pressure and number of wipes currently applied by nurses when using disposable towels during bed baths and propose the minimum values for removing dirt from the skin.

**Methods:**

This multi-study approach consisted of cross-sectional and crossover design components. In Study 1, 101 nurses in two hospitals were observed by recording the wiping pressure and number of wipes when using both disposable (nonwoven) and cotton (woven) towels. Wiping pressure and number of wipes by towel materials were analysed using a linear mixed model. In Study 2, 50 adults received oily and aqueous dirt on their forearms, which were wiped six-times with disposable towels, applying randomly assigned pressure categories. We used colour image analysis and a linear mixed model to estimate the dirt removal rate for each combination of wiping pressure and number of wipes.

**Results:**

Study 1 showed that although wiping pressure did not differ by towel material, the number of wipes was significantly higher for disposable wipes than cotton wipes. Approximately 5% of nurses applied strong wiping pressure or wiped too often. In Study 2, wiping three times with disposable towels at least 5–10 mmHg achieved dirt removal rates of ≥80%.

**Conclusions:**

Some nurses excessively wiped using disposable towels, which might cause skin problems. However, excessive wiping is not required to adequately remove dirt, regardless of the towel material used in various clinical situations. We recommend wiping at 10–20 mmHg of pressure (just like stroking gently) at least three times to improve the quality of bed baths. These findings highlight the need to develop skin-friendly bed bath educational programmes, particularly using appropriate frictional irritation to reduce the risk of skin problems.

**Supplementary Information:**

The online version contains supplementary material available at 10.1186/s12912-022-01162-z.

## Background

The older population is growing rapidly worldwide, and one in six people will be aged ≥65 years by 2050 [1]. With aging, the skin of the older adults becomes more vulnerable and prone to various skin lesions [2]. Thus, the demand for high-quality skin care that guarantees skin integrity, cleanliness, and comfort is expected to increase in various healthcare settings.

Skin care, including bathing, showering, and bed bathing, is a core nursing practice in most hospitals worldwide [3]. Among which, bed baths are the most fundamental way of cleaning the skin of patients who have difficulty bathing or showering. Typically, cotton (woven) towels with water and soap or disposable (nonwoven) towels without water are used [4]. In Europe, at least 15% of people aged ≥65 have severe difficulties in bathing [5], and 15% of Danish patients and 29% of Japanese recipients of in-home care are provided daily bed baths [6, 7]. Moreover, the three goals of improving the quality of skin care, namely skin cleanliness, skin integrity, and comfort enhancement, are interrelated [8]. Removing dirt (including oily dirt such as sebum and old stratum corneum, and aqueous dirt such as sweat [4, 9–11]) from the skin is a prerequisite in achieving these three goals for those receiving bed baths. However, nurses often perform this care based on empirical principles [12], and evidence to establish the best practice is lacking [13].

Given that personal hygiene is a basic human need [14], skin cleanliness (specifically, how to remove dirt from the skin) should be a primary consideration in developing high-quality bed bath methods. The degree of topical dirt removal in bed baths is strongly related to frictional irritation (wiping pressure, WP; and number of wipes, NW [11]). However, excessive frictional irritation decreases patients’ skin barrier function and increases the risk of skin tears. Skin tears are traumatic wounds caused by mechanical forces [15, 16], and are a serious problem causing severe pain, decreased quality of life, and prolonged hospitalization [15]. One of the leading causes of these lesions is extrinsic frictional irritation that occurs while patients are being assisted with activities of daily living [17]. For example, frictional irritation by wiping during bed bath exposes patients at high-risk to skin tears [18–20]. The Japanese Society of Wound, Ostomy, and Continence Management [21] showed that friction during bathing and bed bathing is among the main cause of skin tears in Japan (ranked 4/29: top 4.1%). Therefore, WP and NW during bed baths are essential factors that determine skin cleanliness and integrity. Nursing staff must acquire appropriate techniques that do not cause skin tears [22] and should practice bed baths that keep the body clean while protecting the patient’s vulnerable skin.

A descriptive study of bed baths found that 85% of nurses rubbed vigorously on patients’ skin [12]. Excessively intense WP when using cotton towels was identified as a problem with some nurses [23]. In bed baths with cotton towels, 10–20 mmHg of pressure was sufficient to remove oily and aqueous dirt with three wipes [11]. Furthermore, wiping three times with 23–25 mmHg, which nurses routinely practice, significantly decreased skin barrier function in older patients compared with wiping with 12–14 mmHg [24]. There was no significant difference in the patient satisfaction across the different WPs, and none of the WPs caused discomfort [24]. These results suggest the effectiveness of weak pressure using cotton towels, and evidence regarding optimal frictional irritation during bed baths is accumulating.

In recent years, the use of disposable towels (also known as washing without water) has increased remarkably in the United States and Europe [7, 25, 26]. Disposable wipes have the advantages of hygienic towel management and ease of use compared to cotton wipes. Although the use of cotton and disposable towels has been reported at 71.0% and 12.2%, respectively [27], disposable towels became the first choice in bed baths for infected patients [28] during the COVID-19 pandemic. Moreover, two systematic reviews have shown that disposable wipes are no less effective than cotton wipes in preventing skin lesions and removing bacteria in older adults [13, 26]. Furthermore, disposable wipes have been shown to be a valuable alternative to cotton wipes in terms of comfort of the care recipients [29].

Previous studies on disposable wipes did not consider the effects of frictional irritation, which requires critical evaluation. Generally, disposable towels are thinner than cotton towels and have a different sensation on the user. Considering the surface structure of the towels, cotton towels have bits of thread that protrude (also called as pile) from the main surface, resulting in a rough feeling. In contrast, disposable towels have a smooth surface [30], which may absorb less dirt than cotton towels. Thus, the WP and NW required when disposable towels are used may differ from those needed for cotton wipes. However, the actual conditions in clinical practice remain unclear, and directly transferring the evidence of frictional irritation from cotton wipes to disposable wipes would be inaccurate. Additionally, nurses must remove dirt with minimal frictional irritation to maintain skin cleanliness and integrity. Nevertheless, the WP and NW for bed baths with disposable towels that meet these two requirements are unknown.

Therefore, we aimed to quantify the WP and NW currently applied by nurses during bed baths when using disposable towels and propose the minimum values for removing skin dirt. To achieve this objective, we adopted a multi-study approach based on the following research questions:

1. Study 1: What are the actual conditions of WP and NW when clinical nurses use disposable towels?
2. Study 2: What is the minimal effective WP and NW for removing dirt from the skin during bed baths when using disposable towels?

This multi-study approach allows us in addressing the limited evidence of frictional irritation on the use of disposable towel in the skin care process. Subsequently, this study should provide an optimal bed bath technique when removing dirt from the skin while considering skin integrity and cleanliness.

## Study 1: methods

### Study design, setting, and sample

Study 1 employed a two-centre, descriptive, cross-sectional design. This study was conducted from October to November 2021 at two general hospitals in northern Japan that are of different population. Hospital A had approximately 500 beds and 480 registered nurses, while Hospital B had approximately 170 beds and 60 registered nurses. The eligibility criteria for the participants were as follows: 1) registered nurses with three or more years of clinical practice and 2) experience in bed baths using disposable towels. This study excluded novice and advanced-beginner nurses as we have anticipated that clinical experience would influence bed bath skills; nurses with three or more years of clinical experience were previously classified as competent or above [31]. All 105 nurses who met the eligibility criteria were recruited by the hospital nursing managers (Hospital A, *n* = 53; Hospital B, *n* = 52) and were directly informed of the purpose, methods, and anonymity of the study. Those who signed an informed consent form were included in the study. Overall, 101 nurses (Hospital A, *n* = 50; Hospital B, *n* = 51) agreed to be included in the study. This study was approved by the ethical review board of the affiliated university and participating hospitals (approval no. 21–51). The study is in accordance with the Declaration of Helsinki and Strengthening the Reporting of Observational Studies in Epidemiology (STROBE) guidelines [32] (Additional file 1).

Based on a previous study [23], nurses were asked to wipe the flexed forearm of a simulated patient using disposable towels under three conditions. The application of WP and NW were classified into: A) ordinary (WP and NW applied in daily bed baths); B) weak (WP and NW for patients with vulnerable skin); and C) strong (WP and NW for patients with heavily contaminated skin). We also requested the nurses to perform the same procedure using cotton towels, which will serve as a reference.

### Outcomes

#### Questionnaire on participant characteristics and daily practice of bed baths

A questionnaire was used to investigate the demographic and clinical characteristics of the participants, including age, nursing experience, sex, department, education, professional qualifications, frequency of bed baths, towels mainly used for daily use, skin care learning, experience with skin problems during skin care, and control of WP.

#### WP and NW

WP is defined as the force applied vertically to the skin surface during bed baths [23]. We measured WP using a pressure sensor (SR Soft Vision; Sumitomo Riko Co., Ltd., Nagoya, Japan) [23]. According to previous studies [11, 23, 24], the unit of wiping pressure used was in mmHg (1 mmHg = 101,325/760 Pa [≈133 Pa]). The forearm of the simulated patient (healthy adult) was set on a table at an angle of 30°, imitating the position of a bedridden patient for a bed bath. Nurses were instructed to wipe at least three times, the area from the patient’s wrist to the elbow either peripherally to centrally or a round trip to the back. The average of the three wipes in the central area of the forearm where the pressure was uniformly applied was used for the analysis. A video camera was used to record the NW.

## Study 2: methods

### Study design and setting

Study 2 was a single-blind, crossover, quasi-experimental study in which participants underwent two procedures on the same day in the laboratory. Two types of pseudo-skin dirt, (A) oily and (B) aqueous, were randomly administered to the flexed right and left forearms of each participant, respectively. Each participant was wiped six times using disposable towels with one of four randomly assigned magnitudes of pressure. The block randomization sequence was based on computer-generated random numbers (block size: forearm and dirt type = 4; pressure category = 8). The random assignment was blinded only to the participants. We conducted the study from February to April 2022 in a controlled laboratory at a room temperature of 20–24 °C, 40–60% humidity, and illuminance of 910 lx. This study is in accordance with the Transparent Reporting of Evaluations with Non-randomized Designs (TREND) guidelines [33] (Additional file 2).

### Participants

This study included healthy adults aged ≥20 years. The exclusion criteria were as follows: 1) participants with skin abnormalities or diseases, such as excessive sweating, redness, swelling, or oedema at the forearm; 2) participants with thyroid disease; and 3) participants with a history of skin abnormalities caused by disinfection or cosmetic use. In addition, we requested that the participants avoid the following situations to minimize potential bias: 1) alcohol consumption 8 h prior to study participation; 2) caffeinated beverages and spices on the day of the study; 3) applying moisturizers, sunscreen, or ointments to the forearms on the day of the study; 4) exercise that may cause sweating on the day of the study; and 5) eating 1 h before data collection. Informed consent was obtained from those who indicated willingness to participate in the study. This study was approved by the ethical review board of the affiliated university (approval no. 21–80) in accordance with the Declaration of Helsinki.

The sample size was calculated using G Power software ver. 3.19 [34]. Referring to a previous study on the cleaning effect of cotton wipes [11], the minimum sample size was 48, with an effect size of 0.25, a significance level of 0.05, and a power of 0.80. Therefore, 50 individuals were recruited for this study.

### Interventions

#### Bed bath methods

**Fig. 1:**
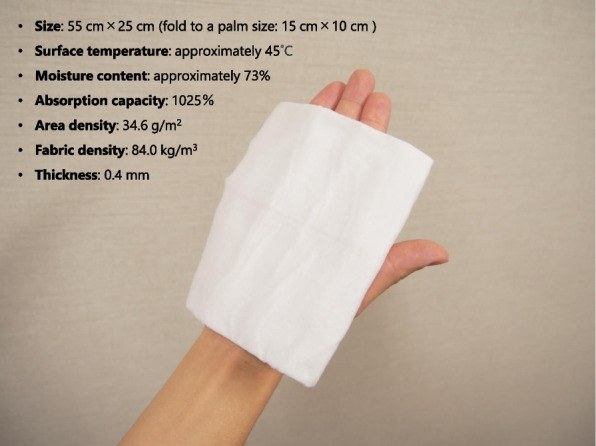
Disposable towel used in this study

Disposable towels (Hakujuji Co., Ltd., Tokyo, Japan) were selected from products that are widely used in clinical settings (Fig. 1). The towels were heated in a microwave to a surface temperature of approximately 45 °C and folded to a palm size. The towels contained the following ingredients: aqua, propylene glycol (humectant), 1,3-butylene glycol (humectant), glycereth-26 (humectant), polyquaternium-51 (humectant), benzalkonium chloride (preservative, disinfectant), iodopropynyl butylcarbamate (preservative), methylisothiazolinone (preservative), cetrimonium chloride (antistatic). The towel did not contain detergents, which did not adversely influence our primary outcome, the dirt removal rate.

WP was measured as described in Study 1. The well-trained researcher wiped six times with disposable towels at one of four randomly assigned pressure categories (WP in mmHg: 5≤WP<10, 10≤WP<20, 20≤WP<30, 30≤WP<40). The range of 5–40 mmHg was determined by the results of Study 1 (range of ordinary condition). The range of wipes was 1–6 times based on the results of a previous study [11] and Study 1 (interquartile range for ordinary condition). We used a new disposable towel for each wipe and wiped the participant’s forearm from the peripheral to the central direction at a speed of 1 time/s.

#### Adhesion of the pseudo-dirt

Previous studies on bed baths have used the microorganism count to indicate skin cleanliness [35, 36]. However, this method is limited such that the degree of contamination prior to data collection cannot be standardized because of the frequency of skin care and associated skin contamination of the patient, which may not allow for precise evaluation. Therefore, methods for intentionally applying dirt and evaluating the removal rate have been used to precisely evaluate the degree of dirt removal based on differences in wiping methods [9, 13]. Thus, this study visualized skin cleanliness using colour image analysis according to previous methods [11]. We used two types of pseudo-skin dirt: (A) an oily lipstick (Lipstick S578 red series; Chifure Holdings Co., Ltd., Kawagoe, Japan) and (B) povidone-iodine (Swabsticks Apliswab 12[LT]; Hakujuji Co., Ltd., Tokyo, Japan). The main composition of this lipstick is wax esters, triglyceride, and oleic acid, like skin surface lipids and sebum. So, previous studies have used this as a model of oily dirt [9, 11]. The povidone-iodine is an aqueous solution commonly used in clinical settings, and its prolonged persistence on the skin causes dermatitis. Therefore, it was used as a model of aqueous dirt that needed to be wiped off [11]. Each skin dirt was applied uniformly over a 9-cm^2^ area at the centre of each flexed forearm (right and left), and the wiping method described above was performed approximately 3 min later.

### Outcomes

#### Participant characteristics

We asked participants to complete questionnaires regarding age, sex, body mass index, and Japanese skin type (JST) during a 15-min resting period before the study began. The JST, a Japanese version of the Fitzpatrick skin phototype [37], was examined to assess whether the participant’s skin colour affected colour image analysis. This scale classifies skin into three types according to susceptibility to sunburn (J-I: sensitive and tanned less than average, J-II: intermediate, and J-III: insensitive and developed a dark tan). In addition, because the participant’s skin barrier function before wiping could influence the results, two representative parameters were measured according to previous guidelines [38]. First, the transepidermal water loss was measured using a VapoMeter SWL5001 (Delfin Technologies Ltd., Kuopio, Finland) for approximately 10 s; one single measurement was used for the data. Stratum corneum hydration was measured using a Moisture Meter SC Compact (Delfin Technologies Ltd., Kuopio, Finland). We took three measurements and used the average value as the data.

#### Dirt removal rate

The wiping area was captured with a digital camera (OLYMPUS PEN Lite E-PL6, Olympus Corporation, Tokyo, Japan) at a fixed height and position before wiping, after applying skin dirt, and after each wipe. The 3 cm central side of the wiping site was also included in the field of photography to confirm the degree of contamination stretched by wiping. We captured the skin images with the same settings for pixel count, shutter speed, diaphragm value, ISO sensitivity, and white balance as in a previous study [11]. Moreover, we used a colour chart (CASMATCH, BEAR Medic Corporation, Kuji, Japan) and Adobe Photoshop (Adobe Inc., San Jose, CA, USA) for skin image colour standardization and size correction.

We calculated the skin dirt removal rates (%) from images captured before and after each wipe using colour analysis software (Feelimage Analyzer; VIVA Computer Inc., Osaka, Japan). The software description and details of the formula for calculating dirt removal rates are shown in a previous study that developed digital image colour analysis [11]. In this method, a dirt removal rate of ≥80% can be considered as sufficient dirt removal.

To obtain clinical reference data for assessing whether the dirt had been sufficiently removed, we calculated the dirt removal rate by washing. After wiping six times, each piece of skin dirt was applied again without overlapping the area to which the dirt was previously applied. Subsequently, following a previous study [39] and a skin care guidebook [40], the washing method recommended for protecting the skin of older adults and removing dirt (after applying foaming body soap, rub with the hands gently clockwise for 30 s at a rate of 1 time/s, and then wash with hot water for 20 s) was performed.

### Statistical analysis

We presented continuous variables as the mean and standard deviation (SD) or median and interquartile range, and categorical variables as the frequency and percentage. All analyses were performed using JMP® 16 Pro (SAS Institute Inc.) with a significance level of 5%.

In Study 1, a linear mixed model with WP and NW as the dependent variable was constructed. This model included nurses as a random effect, and towel material (cotton and disposable towel), condition (strong, ordinary, and weak), and their interaction as a fixed effect. The Bonferroni method for multiple comparisons within conditions was used if any interaction or main effect was significant. In addition, the Wilcoxon signed-rank test was used to compare the towel materials for each condition. The partial *η*^*2*^ and *r* were calculated as effect sizes [41, 42].

Using a separate linear mixed model, Study 2 analysed the relationship between the two types of dirt removal rates and explanatory variables. This model used participants as a random effect, and WP (four categories), NW (six categories), and their interaction as a fixed effect. We then estimated the least squares means (LSMs) of the two types of dirt removal rates for each combination of WP and NW. The relationship between skin barrier function or skin type and the dirt removal rate was confirmed using Spearman’s rank correlation coefficient and one-way ANOVA, respectively. We calculated the intraclass correlation coefficient (ICC) and standard error of measurement to evaluate the intra-rater reliability of the WP measurements. The ICC considered almost perfect reliability to be 0.81–1.00 [43].

## Study 1: results

### Questionnaire on participant characteristics and daily practice for bed baths

The participants’ mean nursing experience was 18.7 (SD, 9.7) years, and 87.1% were female (Table 1). A total of 62.4% belonged to hospital wards and 37.6% belonged to outpatient and other departments. Moreover, 9.9% (*n* = 10/101) of the nurses had experienced skin problems during bed baths, which was more common than in other hygiene care practices. Skin problems during bed baths included redness (36.4%), epidermal peeling (36.4%), skin tears (9.1%), petechia (9.1%), and bleeding due to laceration (9.1%), all of which were reported to have occurred during wiping.

**Table 1:** Participant characteristics and daily practice for bed baths in Study 1 (*n* = 101)

Variables	Values
Age [years]: Mean (SD)	41.6 (10.1)
Nursing experience [years]: Mean (SD)	18.7 (9.7)
Sex: N (%)	
Female	88 (87.1)
Male	13 (12.9)
Department: N (%)	
Hospital wards	63 (62.4)
Outpatient or other	38 (37.6)
Education: N (%)	
Vocational school	90 (89.1)
University	6 (5.9)
Graduate school (Master course)	2 (2.0)
Other	3 (3.0)
Professional qualifications: N (%)	
Yes	15 (14.9)
No	86 (85.1)
Frequency of bed baths: N (%)	
More than 4 times a week	28 (27.7)
3–4 times a week	20 (19.8)
1–2 times a week	11 (10.9)
Rarely	42 (41.6)
Towel used mainly for daily use ^a^: N (%)	
Disposable towel	61 (61.0)
Cotton towel	28 (28.0)
Both	11 (11.0)
Skin care learning: N (%)	
Yes	62 (61.4)
No	39 (38.6)
Experience with skin problems during skin care: N (%)	
Bed bath	10 (9.9)
Bathing	1 (1.0)
Shower	1 (1.0)
Perineal care	1 (1.0)
Other	4 (3.9)
None	84(83.2)
Control of wiping pressure: Disposable wipes: N (%)	
Yes	80 (79.2)
No	6 (5.9)
Not used at all	15 (14.9)
Control of wiping pressure: Cotton wipes: N (%)	
Yes	67 (66.3)
No	2 (2.0)
Not used at all	32 (31.7)

*Notes*: *SD* Standard deviation; a, *n* = 100

### WP and NW by towel material

**Fig. 2:**
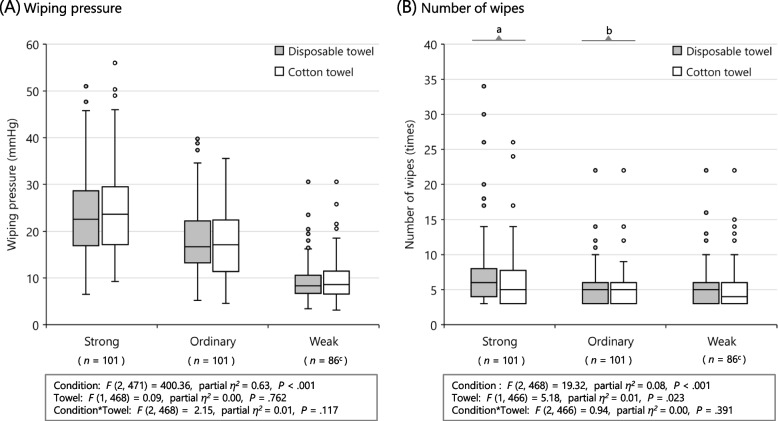
Box plots indicating distributions of wiping pressure and number of wipes by towel materials. *Notes*: Box plots of (**A**) wiping pressure (WP) and (**B**) number of wipes (NW) by towel materials. The median is denoted by the line within the box; 25th percentile, bottom border of box; 75th percentile, top border of box; variability outside the interquartile range, whiskers; and outside values, dots. The nurses applied WP and NW based on three classifications: ordinary (WP and NW applied in daily bed baths); weak (WP and NW for patients with vulnerable skin); and strong (WP and NW for patients with heavily contaminated skin). See Additional file 3 for details of statistical information; Interaction and main effect were analysed in a linear mixed model for two-way repeated-measures ANOVA (Effect size was calculated as partial *η*^*2*^); Wilcoxon signed-rank test showed that the number of wipes using disposable towel was significantly more often than that of cotton towel under the strong (a, *P* < .001) and ordinary (b, *P* = .018) condition. c, approximately 15% of the nurses performed patting only in the weak condition (Cotton towel; *n* = 84)

The box plots in Fig. 2 show the WP and NW used in clinical practices. Approximately 5% of nurses (outliers) applied strong wiping pressure or wiped too often. The details of statistical information are shown in Additional file 3. Four of the ten nurses whose patients had experienced skin problems during bed baths recorded outliers in either WP or NW. Approximately 15% of the nurses performed patting only in the weak condition. In the model for WP, although the main effect of condition was significant (*F*_[2, 471]_ = 400.36, partial *η*^*2*^ = 0.63, *P* < .001), that of towel (*F*_[1, 468]_ = 0.09, partial *η*^*2*^ = 0.00, *P* = .762) and their interaction (*F*_[2, 468]_ = 2.15, partial *η*^*2*^ = 0.01, *P* = .117) was not (Fig. 2[A]). Many nurses responded to the questionnaire claiming that they controlled their WP (Table 1); concordantly, multiple comparisons of condition by towel material showed significant differences for all combinations. The model for NW showed significant effects of condition (*F*_[2, 468]_ = 19.32, partial *η*^*2*^ = 0.08, *P* < .001) and towel type (*F*_[1, 466]_ = 5.18, partial *η*^*2*^ = 0.01, *P* = .023), but their interaction was not significant (*F*_[2, 466]_ = 0.94, partial *η*^*2*^ = 0.00, *P* = .391: Fig. 2[B]). Disposable towels were wiped significantly more often than cotton towels. Owing to an exploratory analysis, no statistically significant associations between WP or NW and the other questionnaire items or hospitals were found.

## Study 2: results

### Participant characteristics and experimental reliability

A total of 50 healthy adults participated in the study with a mean age (SD) of 24.6 (4.5) years, and 58.0% were female. Table 2 shows the participant characteristics from Study 2. No statistically significant associations were observed between skin barrier function, skin type, and dirt removal rate. The ICC of WP for six wipes was 0.990 (95% confidence interval [CI], 0.985–0.994) for the removal of oily dirt and 0.989 (95% CI, 0.984–0.994) for the removal of aqueous dirt, showing almost perfect reliability (Table 3).

**Table 2:** Participant characteristics in Study 2 (*n* = 50)

Variables	Values
Age [years]: Mean (SD)	24.6 (4.5)
Body Mass Index [kg/m^2^]: Mean (SD)	21.0 (2.7)
Sex: N (%)	
Female	29 (58.0)
Male	21 (42.0)
TEWL [g/m^2^/h]: Mean (SD)	
Right	9.6 (2.8)
Left	10.0 (2.9)
SCH [AU]: Mean (SD)	
Right	21.7 (11.2)
Left	22.0 (12.8)
JST: N (%)	
J-1	11 (22.0)
J-2	24 (48.0)
J-3	15 (30.0)

*Notes: JST* Japanese skin type, *SCH* Stratum corneum hydration, *SD* Standard deviation, *TEWL* Transepidermal water loss

**Table 3:** Intra-rater reliability of the wiping pressure performed six times for two types of skin dirt

Skin dirt	SEM	ICC	95%CI		*P*-value
			LL	UL	
Oily	2.57	0.990	0.985	0.994	<.001
Aqueous	2.76	0.989	0.984	0.994	<.001

*Notes*: *Aqueous* Wiping pressure for pseudo-aqueous dirt; *CI* Confidence interval, *ICC* Intraclass correlation coefficients, *LL* Lower limit, *Oily* Wiping pressure for pseudo-oily dirt, *SEM* Standard error of measurement, *UL* Upper limit

### Oily dirt removal rate by wiping with disposable towels

**Fig. 3:**
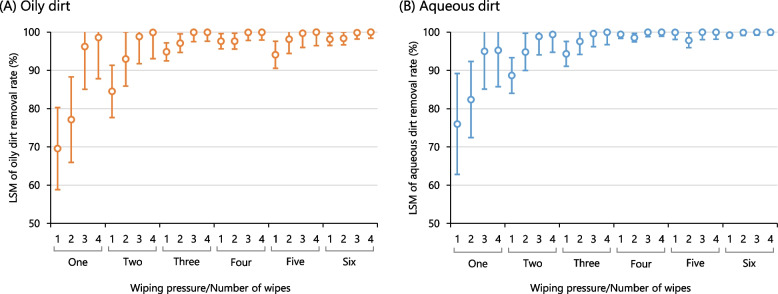
Least squares means (LSMs) of skin dirt removal rate by wiping using disposable towel. *Notes*: LSMs of removal rate for (**A**) oily dirt and (**B**) aqueous dirt by categories of wiping pressures or number of wipes (*n* = 50); In the digital image colour analysis, a dirt removal of rate ≥80% can be considered as a sufficient dirt removal. The circles indicate the LSMs as determined by a linear mixed model as a function of the combination of wiping pressure and number of wipes; The error bars indicate the 95% confidence interval; The wiping pressure (WP: mmHg) is classified into four categories represented by the numbers on the x-axis;1, 5≤WP<10; 2, 10≤WP<20; 3, 20≤WP<30; 4, 30≤WP<40

The LSMs of the oily dirt removal rate estimated using the linear mixed model (Additional file 4) are shown in Fig. 3[A]. The combinations of the NW and WP that achieved an oily dirt removal rate ≥80% (including 95% CI) were as follows: one wipe with 20≤WP<30 and 30≤WP<40; two wipes with 10≤WP<20, 20≤WP<30, and 30≤WP<40; three wipes with 5≤WP<10, 10≤WP<20, 20≤WP<30, and 30≤WP<40. Similarly, four to six wipes showed a sufficient dirt removal rate, regardless of the WP. Furthermore, we confirmed no transition of skin contamination in the wipe direction for any combination (Additional file 5). In contrast, the oily removal rate was 13.0% (95% CI: 7.2–18.9%) when washing with foaming soap and warm water.

### Aqueous dirt removal rate by wiping with disposable towels

The LSMs of the aqueous dirt removal rate estimated using the linear mixed model (Additional file 4) are shown in Fig. 3[B]. The combinations of the NW and WP that achieved an aqueous dirt removal rate of ≥80% were as follows: one wipe with 20≤WP<30 and 30≤WP<40; two wipes with 5≤WP<10, 10≤WP<20, 20≤WP<30, and 30≤WP<40. Similarly, wiping three to six times resulted in a sufficient dirt removal rate regardless of the WP. There was no transition of skin contamination in the wipe direction (Additional file 5). The removal rate after washing was 100% for all the participants.

### Integration of the results of studies 1 and 2

**Fig. 4:**
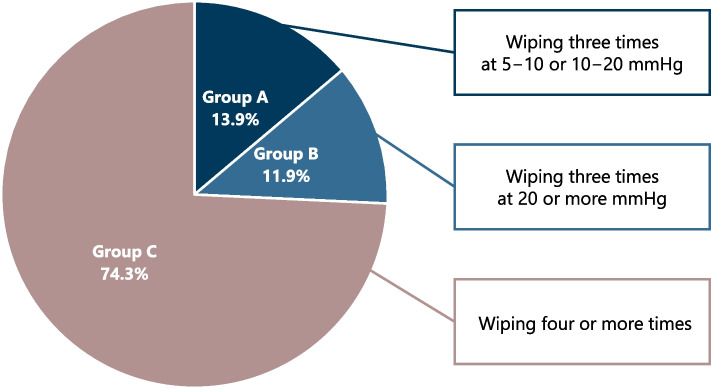
Nurses who performed the minimal effective wiping pressure and number of wipes during disposable wipes. *Notes*: Group A (*n* = 14), the group of nurses who performed the minimal effective wiping pressure and number of wipes (i.e., wiping three times at 5–10 or 10–20 mmHg); Group B (*n* = 12), the group of nurses who wiped three times at 20 or more mmHg; and Group C (*n* = 75), the group of nurses who wiped four or more times

In Study 1, 13.9% (*n* = 14/101) of nurses practiced wiping three times at 5–10 (*n* = 2) or 10–20 (*n* = 12) mmHg during disposable wipes (Fig. 4).

## Discussion

### WP and NW applied by nurses during bed baths using disposable towels

To the best of our knowledge, Study 1 was the first cross-sectional study to visualize WP and NW applied by the clinical nurses when using disposable towels during bed baths. We expected that the WP would differ between disposable and cotton towels according to the surface, thinness, and sensation felt by nurses during use. However, no statistically significant differences were observed for each pressure condition. As most nurses reported that they controlled their WP in their daily practice, multiple comparisons of WP by towel material showed significant differences in all combinations. In other words, nurses could control the WP according to different patient situations, regardless of the towel material.

Interestingly, the NW was higher for disposable wipes than cotton wipes, although the effect size was relatively small. Disposable towels generally have smooth surfaces without piles [30]. Thus, nurses may wipe more due to potential perceptions that disposable wipes require more effort to remove dirt. Previous studies have also reported that some care recipients perceive that the use of cotton towels with water and soap more effectively removes dirt and makes them feel “really” cleaner than disposable wipes [7, 29, 44]. Therefore, we inferred that the nurses in this study had a similar perception, which was evident in their practice (i.e., NW).

We found that approximately 10% of the nurses had experienced skin problems such as skin tears due to wiping, 40% of whom were outliers in either WP or NW. Although most nurses controlled WP, the tendency for some nurses to apply excessive WP was consistent with a prior study [23]. In particular, many outliers under weak conditions were observed regardless of the towel material. This indicates that some nurses apply strong WP or wipe too often and perceive it as weak friction irritation. Such inappropriate practices [16, 22] may lead to skin problems during bed baths. Although a previous study did not distinguish between the prevalence of skin tears by bathing and bed bathing [21], our results showed that frictional irritation by bed bathing may be more related to skin problems than bathing. These results reaffirm the value of our research, which aimed to identify the optimal frictional irritation during bed baths to reduce patient risk.

### Combination of minimum WP and NW to remove skin dirt with disposable towels

Study 1 was used to determine the methodology and interpret the results of Study 2. The current study revealed that wiping three times with 5–10 mmHg using disposable towels was sufficient to remove oily and aqueous skin dirt. For robust oily dirt, the 95% CI of the removal rate exceeded 80% by wiping at ≥10 mmHg, even with only two wipes. However, the estimated accuracy was higher with three wipes than two, suggesting that wiping at least three times with a disposable towel is desirable. Furthermore, this minimum combination may apply to the older adults, as our results indicated that the cleaning effect of wiping was higher than that of the washing method recommended for older patients.

Wiping three times at 10–20 mmHg using cotton towels can sufficiently remove skin dirt while maintaining skin barrier function of older patients [11, 24]. We expected that the cleaning effect of disposable wipes might be lower than that of cotton wipes owing to differences in towel surface construction; however, the results did not support this hypothesis. The need to wipe three times was consistent with a previous study [11], but disposable wipes (5–10 mmHg) could remove oily dirt with a weaker WP than cotton wipes (10–20 mmHg). While cotton towels have piles creating a small gap between the skin surface and the towel when the WP is weak, disposable towels have a smooth surface, which allows the towel to adhere to the skin surface even when the WP is weak. Thus, nurses can suggest that patients use disposable towels when their skin is highly contaminated by oily dirt, diarrhoea, or excessive sweating.

This study was consistent with a previous study [11] in that both types of towels could adequately remove dirt by wiping three times with 10–20 mmHg despite the structural differences between the two towels. A phenomenological qualitative study by Veje et al. [7] reported that hospitalized patients generally preferred cotton wipes, but preferred disposable wipes under certain circumstances, such as pain and diarrhoea. They also recommended that nurses acknowledge and incorporate patients’ preferences in decisions regarding the appropriate bed bath method [7, 45]. In line with these results, our findings could aid in performing bed baths with respect to patients’ preferences of towels. Moreover, the educational advantage of teaching evidence-based WP techniques, regardless of the towel material, could contribute to their application in clinical practice.

Overall, we suggest that nursing staff do not need to excessively wipe with disposable towels during bed baths. Instead, they can adequately remove dirt from the skin by wiping three times with weak pressure. In Study 1, 13.9% (*n* = 14/101) of nurses applied wiping three times at 5–10 or 10–20 mmHg (Fig. 4), indicating that few nurses performed the appropriate practice during bed baths with disposable towels. Therefore, these findings are vital for clinical practice and could reduce harm to patients, and thus lead to desirable bed bath practices in terms of skin integrity and cleanliness.

### Study limitations

This study had several limitations. First, the generalizability of the bed bath methods reported in Study 1 is limited because the simulated patients were standardised and first- and second-year nurses were excluded. This was unavoidable because we prioritized the reliability and validity of the WP measurements. We encouraged the nurses to wipe as usual to reflect the actual clinical situation in our data. Second, whether the proposed combination has a similar cleaning effect on the skin of babies, children, and older adults is uncertain. The skin barrier undergoes a period of optimization following birth [46]. Children have a highly permeable skin barrier, which matures to the same level as adults when they are approximately 5 years old [47]. With aging, the skin of older adults becomes more vulnerable [2, 46]. Therefore, further studies on friction irritation due to disposable wipes in populations with vulnerable skin (babies, children under five, and older adults) are needed.

## Conclusions

This study determined the WP and NW currently applied by the nurses when using disposable towels during bed baths and proposed the minimum values that can remove skin dirt. In Study 1, the WP of disposable wipes and cotton wipes did not differ, but the NW was higher for disposable wipes than for cotton wipes. Some nurses apply strong WP or wipe too often, which could lead to skin problems such as skin tears. Study 2 quantitatively indicated that wiping using disposable towels at a minimum of three times, with a 5–10 mmHg of pressure was adequate to remove oily and aqueous skin dirt. In conclusion, nurses do not need to excessively wipe using disposable towels to adequately remove skin dirt in bed baths. Considering the cleaning effect of cotton wipes, we recommend wiping at least three times with weak pressure (10–20 mmHg).

### Relevance to clinical practice

Clinical nursing staff must practice bed baths that keep the body clean while protecting vulnerable skin. However, the decision to use disposable or cotton towels may be influenced by patient preferences and differences in hospital philosophy, financial situation, and international background. This study suggests that nurses can adequately remove dirt by wiping at least three times with a 10–20 mmHg of pressure (“wiping with a towel just like stroking gently against the skin surface [24]”), using either type of towel in various clinical situations. Additionally, nurses and patients could select a disposable towel, which has a higher cleaning effect even when wiped at a weaker pressure (5–10 mmHg), or when the patient’s skin is highly contaminated with oily dirt, diarrhoea, or excessive sweating. Finally, this study provides a framework for future education on friction irritation during bed baths. Developing skin-friendly bed bath educational programmes for nurses and caregivers on the appropriate application of WP and NW is expected to reduce the risk of skin problems such as skin tears.

## Supplementary Information

**Additional file 1.** STROBE statement checklist.

**Additional file 2.** TREND statement checklist.

**Additional file 3.** The details of statistical information for wiping pressure and number of wipes by towel materials.

**Additional file 4.** The linear mixed model for skin dirt removal rate.

**Additional file 5.** The transition of skin contamination in the wipe direction.

## Data Availability

The datasets generated and/or analysed during the current study are not publicly available due to ethical restrictions or confidentiality of participants but are available from the corresponding author on reasonable request.

## Abbreviations

CI: Confidence interval
ICC: Intraclass correlation coefficient
JST: Japanese skin type
LSM: Least squares mean
NW: Number of wipes
SD: Standard deviation
WP: Wiping pressure
