# Supplementary material for: Minimum wiping pressure and number of wipes that can remove dirt during bed baths using disposable towels: a multi-study approach
Source: BMC Nurs. 2023 Jan 16;22:18. doi: 10.1186/s12912-022-01162-z (PMC9842401; doi:10.1186/s12912-022-01162-z)
Supplement: Supplementary file 3 — Additional file 3. The details of statistical information for wiping pressure and number of wipes by towel materials. [file 12912_2022_1162_MOESM3_ESM.docx]

**Additional file 3.** The details of statistical information for wiping pressure [WP] and number of wipes [NW] by towel materials.

|  | Condition ^c^ | | | Main effect | | | | | | Interaction | | |
| --- | --- | --- | --- | --- | --- | --- | --- | --- | --- | --- | --- | --- |
|  | Strong | Ordinary | Weak | Condition | | | Towel | | | Condition × Towel | | |
|  | (n = 101) | (n = 101) | (n = 86 ^d^) | F (df) | partial η^2^ | P ^a^ | F (df) | partial η^2^ | P ^a^ | F (df) | partial η^2^ | P ^a^ |
| **Wiping pressure (mmHg)** | | ** |  | 400.36  (2, 471) | 0.63 | ＜.001 | 0.09  (1, 468) | 0.00 | .762 | 2.15  (2, 468) | 0.01 | .117 |
| Disposable towel |  |  |  |  |  |  |  |  |  |  |  |  |
| Mdn (IQR) | 22.6 (16.9-28.6) | 16.7 (13.2-22.2)  ** | 8.3 (6.7-10.6)  ** |  |  |  |  |  |  |  |  |  |
| M (SD) | 23.5 (9.2) | 18.6 (7.6)  ** | 9.5 (4.6) |  |  |  |  |  |  |  |  |  |
| Cotton towel |  | ** |  |  |  |  |  |  |  |  |  |  |
| Mdn (IQR) | 23.7 (17.1-29.5) | 17.1 (11.4-22.4)  ** | 8.6 (6.6-11.5) |  |  |  |  |  |  |  |  |  |
| M (SD) | 24.4 (9.6) | 17.6 (7.4) | 9.9 (5.4) |  |  |  |  |  |  |  |  |  |
| r-value | 0.15 | 0.14 | 0.02 |  |  |  |  |  |  |  |  |  |
| P-value ^b^ | .143 | .172 | .881 |  |  |  |  |  |  |  |  |  |
| **Number of wipes (times)** | |  |  | 19.32  (2, 468) | 0.08 | ＜.001 | 5.18  (1, 466) | 0.01 | .023 | 0.94  (2, 466) | 0.00 | .391 |
| Disposable towel | ** | ** |  |  |  |  |  |  |  |  |  |  |
| Mdn (IQR) | 6.0 (4.0-8.0) | 5.0 (3.0-6.0) | 5.0 (3.0-6.0) |  |  |  |  |  |  |  |  |  |
| M (SD) | 7.0 (5.3) | 5.6 (3.0) | 5.5 (3.1) |  |  |  |  |  |  |  |  |  |
| Cotton towel |  | *  ** |  |  |  |  |  |  |  |  |  |  |
| Mdn (IQR) | 5.0 (3.0-7.8) | 5.0 (3.0-6.0) | 4.0 (3.0-6.0) |  |  |  |  |  |  |  |  |  |
| M (SD) | 6.3 (4.6) | 5.3 (2.9) | 5.2 (3.3) |  |  |  |  |  |  |  |  |  |
| r-value | 0.36 | 0.23 | 0.21 |  |  |  |  |  |  |  |  |  |
| P-value ^b^ | ＜.001 | .018 | .051 |  |  |  |  |  |  |  |  |  |

**Notes**: df, degree of freedom; M (SD), mean (standard deviation); Mdn (IQR), median (interquartile range); The nurses wiped with three conditions of WP and NW: ordinary (WP and NW applied in daily bed baths); weak (WP and NW for patients with vulnerable skin); and strong (WP and NW for patients with heavily contaminated skin).

a, Interaction and main effect were analysed in a linear mixed model for two-way repeated-measures ANOVA (Effect size was calculated as partial η^2^)

b, Wilcoxon signed-rank test (Effect size was calculated as r)

c, Bonferroni method for multiple comparisons of pressure condition by each towel material (*, P ＜ .05; **, P ＜ .01)

d, Approximately 15% of the nurses performed patting only in the weak condition (Cotton towel; n = 84).
