# Supplementary material for: Minimum wiping pressure and number of wipes that can remove dirt during bed baths using disposable towels: a multi-study approach
Source: BMC Nurs. 2023 Jan 16;22:18. doi: 10.1186/s12912-022-01162-z (PMC9842401; doi:10.1186/s12912-022-01162-z)
Supplement: Supplementary file 4 — Additional file 4. The linear mixed model for skin dirt removal rate. [file 12912_2022_1162_MOESM4_ESM.docx]

**Additional file 4.** The linear mixed model for skin dirt removal rate (n = 50)

|  | Oily dirt removal rate | | | | Aqueous dirt removal rate | | | |
| --- | --- | --- | --- | --- | --- | --- | --- | --- |
|  | Estimate | SE | t-value | P-value | Estimate | SE | t-value | P-value |
| Constant | 95.528 | 0.928 | 102.950 | <.001 | 96.548 | 0.649 | 148.850 | <.001 |
| Number of wipes |  |  |  |  |  |  |  |  |
| One | -10.162 | 1.892 | -5.370 | <.001 | -9.367 | 1.739 | -5.390 | <.001 |
| Two | -1.473 | 0.930 | -1.580 | .120 | -1.084 | 0.751 | -1.440 | .269 |
| Three | 2.424 | 0.634 | 3.820 | <.001 | 1.337 | 0.575 | 2.330 | .529 |
| Four | 3.229 | 0.641 | 5.030 | <.001 | 2.956 | 0.564 | 5.240 | .001 |
| Five | 2.440 | 1.038 | 2.350 | .023 | 2.911 | 0.748 | 3.890 | .005 |
| Six | Reference |  |  |  | Reference |  |  |  |
| Wiping pressure |  |  |  |  |  |  |  |  |
| 5≤WP<10 | -5.741 | 1.586 | -3.62 | <.001 | -3.595 | 1.181 | -3.04 | .036 |
| 10≤WP<20 | -1.986 | 1.629 | -1.22 | .229 | -1.352 | 1.113 | -1.22 | .289 |
| 20≤WP<30 | 3.536 | 1.629 | 2.17 | .035 | 2.377 | 1.113 | 2.14 | .097 |
| 30≤WP<40 | Reference |  |  |  | Reference |  |  |  |
| Number of wipes* Wiping pressure |  |  |  |  |  |  |  |  |
| One* 5≤WP<10 | -10.057 | 3.233 | -3.11 | .003 | -7.580 | 3.606 | -2.1 | .045 |
| One* 10≤WP<20 | -6.274 | 3.321 | -1.89 | .065 | -3.434 | 2.808 | -1.22 | .232 |
| One* 20≤WP<30 | 7.316 | 3.321 | 2.2 | .033 | 5.474 | 2.808 | 1.95 | .062 |
| Two* 5≤WP<10 | -3.802 | 1.589 | -2.39 | .021 | -3.183 | 1.346 | -2.36 | .123 |
| Two* 10≤WP<20 | 0.905 | 1.632 | 0.55 | .582 | 0.728 | 1.296 | 0.56 | .624 |
| Two* 20≤WP<30 | 1.250 | 1.632 | 0.77 | .448 | 1.068 | 1.296 | 0.82 | .486 |
| Three* 5≤WP<10 | 2.617 | 1.084 | 2.42 | .020 | 0.045 | 1.064 | 0.04 | .980 |
| Three* 10≤WP<20 | 1.145 | 1.113 | 1.03 | .309 | 1.056 | 0.980 | 1.08 | .663 |
| Three* 20≤WP<30 | -1.564 | 1.113 | -1.41 | .167 | -0.643 | 0.980 | -0.66 | .752 |
| Four* 5≤WP<10 | 4.592 | 1.096 | 4.19 | <.001 | 3.556 | 1.047 | 3.4 | .011 |
| Four* 10≤WP<20 | 0.826 | 1.126 | 0.73 | .467 | 0.407 | 0.961 | 0.42 | .684 |
| Four* 20≤WP<30 | -2.407 | 1.126 | -2.14 | .038 | -1.888 | 0.961 | -1.97 | .090 |
| Five* 5≤WP<10 | 1.859 | 1.773 | 1.05 | .300 | 4.097 | 1.342 | 3.05 | .017 |
| Five* 10≤WP<20 | 2.134 | 1.821 | 1.17 | .247 | -0.232 | 1.291 | -0.18 | .862 |
| Five* 20≤WP<30 | -1.811 | 1.821 | -0.99 | .325 | -1.836 | 1.291 | -1.42 | .195 |
| Six* 30≤WP<40 | Reference |  |  |  | Reference |  |  |  |

**Notes**: SE = standard error; WP, wiping pressure expressed in mmHg
