# Supplementary material for: Minimum wiping pressure and number of wipes that can remove dirt during bed baths using disposable towels: a multi-study approach
Source: BMC Nurs. 2023 Jan 16;22:18. doi: 10.1186/s12912-022-01162-z (PMC9842401; doi:10.1186/s12912-022-01162-z)
Supplement: Supplementary file 5 — Additional file 5. The transition of skin contamination in the wipe direction. [file 12912_2022_1162_MOESM5_ESM.docx]

**Additional file 5.** The transition of skin contamination in the wipe direction

|  | Oily dirt transition rate (%) | | | | | | Aqueous dirt transition rate (%) | | | | | |
| --- | --- | --- | --- | --- | --- | --- | --- | --- | --- | --- | --- | --- |
|  | One | Two | Three | Four | Five | Six | One | Two | Three | Four | Five | Six |
| 5≤WP<10 |  |  |  |  |  |  |  |  |  |  |  |  |
| ID：42 | 0 | 0 | 0 | 0 | 0 | 0 | 0 | 0 | 0 | 0 | 0 | 0 |
| ID：12 | 0 | 0 | 0 | 0 | 0 | 0 | 0 | 0 | 0 | 0 | 0 | 0 |
| ID：38 | 0 | 0 | 0 | 0 | 0 | 0 | 0 | 0 | 0 | 0 | 0 | 0 |
| ID：8 | **2** | 0 | 0 | 0 | 0 | 0 | 0 | 0 | 0 | 0 | 0 | 0 |
| ID：28 | 0 | 0 | 0 | 0 | 0 | 0 | 0 | 0 | 0 | 0 | 0 | 0 |
| 10≤WP<20 |  |  |  |  |  |  |  |  |  |  |  |  |
| ID：23 | 0 | 0 | 0 | 0 | 0 | 0 | 0 | 0 | 0 | 0 | 0 | 0 |
| ID：14 | 0 | 0 | 0 | 0 | 0 | 0 | 0 | 0 | 0 | 0 | 0 | 0 |
| ID：9 | 0 | 0 | 0 | 0 | 0 | 0 | 0 | 0 | 0 | 0 | 0 | 0 |
| ID：26 | 0 | 0 | 0 | 0 | 0 | 0 | 0 | 0 | 0 | 0 | 0 | 0 |
| ID：44 | 0 | 0 | 0 | 0 | 0 | 0 | 0 | 0 | 0 | 0 | 0 | 0 |
| 20≤WP<30 |  |  |  |  |  |  |  |  |  |  |  |  |
| ID：15 | **4** | 0 | 0 | 0 | 0 | 0 | 0 | 0 | 0 | 0 | 0 | 0 |
| ID：31 | 0 | 0 | 0 | 0 | 0 | 0 | 0 | 0 | 0 | 0 | 0 | 0 |
| ID：24 | 0 | 0 | 0 | 0 | 0 | 0 | 0 | 0 | 0 | 0 | 0 | 0 |
| ID：40 | 0 | 0 | 0 | 0 | 0 | 0 | 0 | 0 | 0 | 0 | 0 | 0 |
| ID：46 | 0 | 0 | 0 | 0 | 0 | 0 | 0 | 0 | 0 | 0 | 0 | 0 |
| 30≤WP<40 |  |  |  |  |  |  |  |  |  |  |  |  |
| ID：7 | **2** | 0 | 0 | 0 | 0 | 0 | 0 | 0 | 0 | 0 | 0 | 0 |
| ID：48 | 0 | 0 | 0 | 0 | 0 | 0 | 0 | 0 | 0 | 0 | 0 | 0 |
| ID：37 | **1** | 0 | 0 | 0 | 0 | 0 | 0 | 0 | 0 | 0 | 0 | 0 |
| ID：30 | 0 | 0 | 0 | 0 | 0 | 0 | 0 | 0 | 0 | 0 | 0 | 0 |
| ID：11 | 0 | 0 | 0 | 0 | 0 | 0 | 0 | 0 | 0 | 0 | 0 | 0 |

**Notes**: Bold is shown that the transition of skin contamination in the wipe direction occurred; Five participants were randomly extracted from each wiping pressure categories.

WP, wiping pressure expressed in mmHg.
